# CDR-Informed Graph Neural Network for Plasma Metabolomics in Lung Cancer and Pulmonary Neuroendocrine Neoplasms

**DOI:** 10.3390/ijms27188119

**Published:** 2026-09-12

**Authors:** Eyad Himdiat, Jean-François Haince, Rashid A. Bux, Guoyu Huang, Paramjit S. Tappia, Bram Ramjiawan, Maria Vaida

**Affiliations:** 1 School of Analytics & Computational Sciences, Harrisburg University of Science and Technology, Harrisburg, PA 17101, USA; ehimdiat@my.harrisburgu.edu; 2 BioMark Diagnostic Solutions Inc., Quebec, QC G1P 4P5, Canada; jhaince@biomarkdiagnostics.com (J.-F.H.); ghuang@biomarkdiagnostics.com (G.H.); 3 BioMark Diagnostics Inc., Richmond, BC V6X 2W2, Canada; rahmed@biomarkdiagnostics.com; 4 Department of Food and Human Nutritional Sciences, Faculty of Agricultural and Food Sciences, University of Manitoba, Winnipeg, MB R3T 2N2, Canada; ptappia@sbrc.ca (P.S.T.); bramjiawan@sbrc.ca (B.R.); 5 Department of Pharmacology & Therapeutics, Max Rady College of Medicine, University of Manitoba, Winnipeg, MB R3T 2N2, Canada

**Keywords:** graph neural network, plasma metabolomics, lung cancer detection, pulmonary neuroendocrine tumor, GraphSAGE, heterogeneous graph, liquid biopsy

## Abstract

Plasma metabolomics offers a promising avenue for the non-invasive classification of lung cancer. However, most classification frameworks treat lung cancer as a single entity, conflating non-small-cell lung cancer (NSCLC) with pulmonary neuroendocrine neoplasms (NENs), despite their distinct biology and treatment pathways. We apply a heterogeneous graph neural network to a three-class problem discriminating Control, NSCLC, and NEN from targeted plasma metabolomic profiling in 800 participants (466 NSCLC, 120 NEN, 214 Controls). Matched metabolites were annotated by Cell Danger Response (CDR) relevance class and expected direction of change, with these annotations encoded as metabolite features and direction-aware patient–metabolite edge weights. Across ten random seeds, the three-class model achieved a macro-F1 of 0.898±0.026 and macro-AUROC of 0.962±0.016. Collapsing the three-class posteriors to a cancer-versus-control score yielded an AUROC of 0.951±0.017, a sensitivity of 0.960±0.011, and an F1 score of 0.949±0.010. A separate cancer-only classifier distinguished NEN from NSCLC with an accuracy of 0.960±0.026 and an AUROC of 0.985±0.020. The gradient-times-input attribution identified one-carbon, glycine–serine, proline, and ornithine–arginine metabolic programs, with proline, C5DC, uric acid, and fumaric acid among the leading annotated metabolites. Removal of all 11 cohort-derived Class N annotations left NSCLC-versus-NEN AUROC essentially unchanged, and broader comparator analyses showed no measurable predictive advantage of the CDR prior over the otherwise matched concentration-weighted GNN. These findings indicate that predictive discrimination is driven primarily by the measured metabolomic features, whereas the CDR component provides a biologically structured framework for directional metabolite and pathway interpretation. Independent external and prospective validation is required to establish generalizability and clinical utility.

## 1. Introduction

Cancer treatment has evolved from conventional cytotoxic approaches toward an increasingly diverse therapeutic landscape that includes targeted therapies, immunotherapies, and other biologically informed strategies [1]. Treatment outcomes remain strongly influenced by the timing and biological characterization of the disease. A central determinant of lung cancer survival is stage at diagnosis. Five-year survival for Stage I non-small-cell lung cancer (NSCLC) exceeds 73%, whereas five-year survival for Stage IV disease is less than 13% [2]. Most cases are detected at advanced stages because lung cancer is often asymptomatic until local or distant invasion is established. Although low-dose computed tomography has demonstrated mortality benefit, its clinical utility is limited by a high false-positive rate that can lead to unnecessary, invasive follow-up procedures. Liquid biopsy approaches that quantify systemic metabolic perturbation in peripheral blood offer a complementary, non-invasive triage tool that is independent of imaging resolution and does not require bronchoscopic or percutaneous sampling [3].

Plasma metabolomics is particularly well suited to this problem because lung tumors reprogram host systemic metabolism through mechanisms including the Warburg effect, altered one-carbon transfer and methyl group utilization, disruption of TCA cycle intermediates, and upregulation of phosphatidylcholine biosynthesis for membrane production in rapidly dividing cells [4]. These perturbations are detectable in peripheral plasma through quantitative targeted profiling of amino acids, organic acids, acylcarnitines, biogenic amines, glycerophospholipids, and sphingolipids using validated HPLC-MS/MS platforms. Haince et al. demonstrated that a panel of plasma metabolites profiled by this approach could achieve high-sensitivity detection of early-stage lung cancer [5].

A distinct diagnostic challenge arises from pulmonary neuroendocrine neoplasms (NENs), which account for approximately 20–25% of all primary lung malignancies but originate from a fundamentally different cellular lineage than NSCLC [6]. Lung neuroendocrine tumors are a heterogeneous group of pulmonary tumors that originate from the neuroendocrine cells of the respiratory tract, historically linked to Kulchitsky cells in the bronchial epithelium. They include typical carcinoid, atypical carcinoid, large-cell neuroendocrine carcinoma, and small-cell lung carcinoma, which differ in grade, aggressiveness, mitotic activity, necrosis, and prognosis [7]. This distinct origin confers distinct metabolic programming. Boullier et al. reported alterations in fatty acid biosynthesis and beta-oxidation, the Warburg effect, and the citric acid cycle across pulmonary neuroendocrine neoplasms, together with subtype-specific changes in individual metabolites such as the serotonin catabolite 5-hydroxyindoleacetic acid and several lysophosphatidylcholines, and showed that these plasma profiles distinguish neuroendocrine neoplasms from both healthy individuals and NSCLC [6].

Clinical management of neuroendocrine neoplasms differs substantially from that of conventional NSCLC. For well-differentiated, somatostatin-receptor-positive neuroendocrine tumors, somatostatin analogues are commonly used for symptom control and tumor-growth inhibition, while peptide receptor radionuclide therapy and targeted agents such as mTOR inhibitors are used in selected advanced or progressive cases [8]. In contrast, NSCLC treatment is guided by histology, stage, PD-L1 expression, and molecular subtype. Patients without actionable driver alterations are commonly treated with platinum-based chemotherapy, often combined with immune checkpoint inhibitors, whereas tumors harboring actionable alterations such as EGFR mutations or ALK, ROS1, RET, NTRK, BRAF, MET, HER2, or KRAS G12C alterations may receive matched targeted therapies [9]. Pre-biopsy identification of a neuroendocrine metabolic signature could help prioritize biopsy strategy, immunohistochemical staining, and specialist referral while tissue confirmation is pursued.

Standard machine learning approaches applied to metabolomics data, including random forests, support vector machines, and regularized regression, treat metabolites as independent features and discard relational information about their co-regulation, shared pathway membership, and documented disease associations. Graph neural networks (GNNs) offer a representational alternative in which such relational structure is encoded directly in the topology and edge weights of a heterogeneous attributed graph, allowing message-passing layers to propagate biologically contextualized representations across patient, metabolite, and disease nodes during training [10,11,12].

Our previously published M-GNN framework demonstrated that heterogeneous metabolite, pathway, and disease relationships could be incorporated into a graph neural network for binary lung-cancer-versus-control classification [13]. However, that framework was limited to the broader binary cancer-detection task, did not explicitly encode the direction of disease-associated metabolite alterations, and was not designed to distinguish conventional NSCLC from pulmonary neuroendocrine neoplasms.

The present work introduces a three-class GNN framework that simultaneously discriminates Control, NSCLC, and NEN classes from plasma metabolomics by augmenting the edge-weighting scheme with a CDR annotation layer that encodes the NEN metabolic panel directly into the graph topology. Stage 1 of the model generates a malignancy probability for all patients using the three-class GNN model. A separate Stage 2 analysis trains a binary classifier on the cancer-only cohort to discriminate NEN from NSCLC.

The three-class model attains a macro-F1 of 0.898±0.026 and a macro-AUROC of 0.962±0.016 across ten random seeds. A separate cancer-only classifier distinguishes NEN from NSCLC with an AUROC of 0.985±0.020. The gradient-times-input attribution identifies one-carbon and glycine–serine metabolic signatures, with proline, C5DC, uric acid, and fumaric acid among the leading annotated metabolites. Matched comparator and ablation analyses, however, do not show a measurable predictive advantage of the CDR prior over the otherwise matched, concentration-weighted GNN. Accordingly, the contribution of the CDR component is interpreted primarily as biological structuring of metabolite and pathway attribution.

## 2. Results and Discussion

### 2.1. Cohort Characteristics and Statistical Comparison

Baseline demographic and clinical characteristics were summarized separately for the Control, NSCLC, and NEN groups. Continuous variables were reported as the mean ± standard deviation, and categorical variables were reported as number and percentage (Table 1). Pairwise standardized mean differences (SMDs) were calculated for Control versus NSCLC, Control versus NEN, and NSCLC versus NEN to quantify the baseline imbalance independently of sample size. Stage information was summarized separately for NSCLC because comparable stage information was not available across the NEN and Control groups.

### 2.2. Three-Class Discrimination of Control, NSCLC, and NEN

Across the ten fixed random seeds, the CDR-informed M-GNN separated the three diagnostic classes with a mean accuracy of 0.904±0.021 and a macro-averaged F1 of 0.898±0.026 on the held-out test partitions (Table 2). Discrimination was strong on threshold-independent measures, with a macro-averaged AUROC of 0.962±0.016 and an AUPRC of 0.940±0.028, and the Matthews correlation coefficient of 0.831±0.039 confirmed that performance was not an artifact of the class imbalance, since this coefficient is computed over all three classes jointly and penalizes majority-class bias. Balanced accuracy (0.896±0.036) tracked raw accuracy closely, indicating that the minority neuroendocrine class was not sacrificed to inflate the overall score.

**Figure 1:**
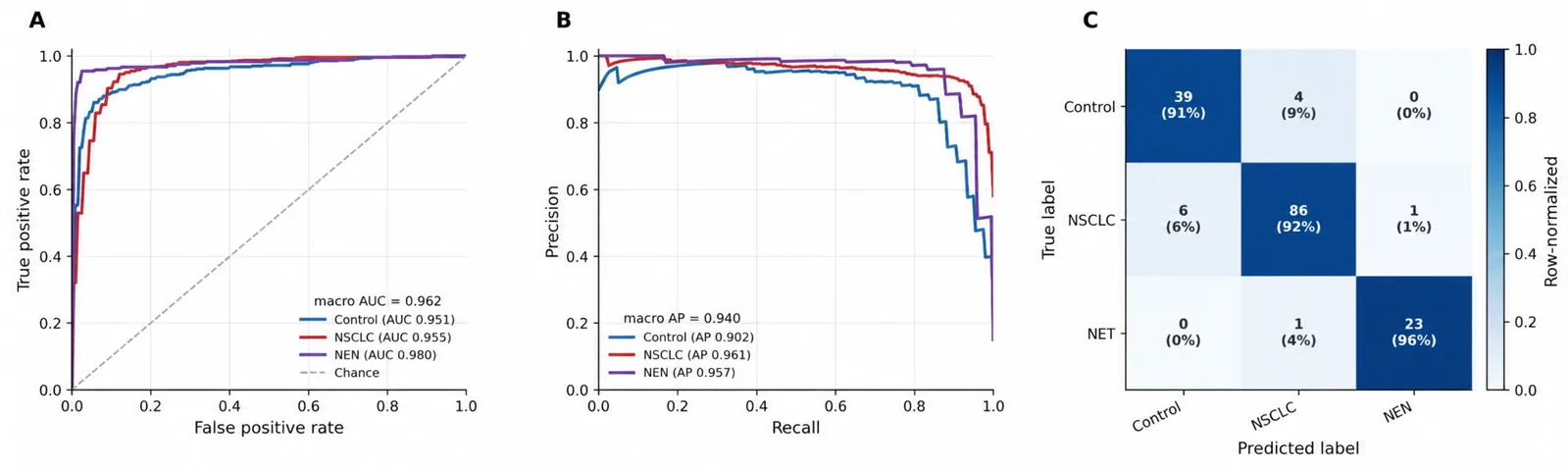
Three-class (Control/NSCLC/NEN) performance of the CDR-informed M-GNN on held-out test patients. (**A**) One-versus-rest receiver operating characteristic curves, showing the highest AUROC for the NEN class. (**B**) Precision–recall curves for the three diagnostic classes. (**C**) Row-normalized confusion matrix for the best-performing seed, showing that most residual misclassification arises from Control patients predicted as NSCLC.

The per-class breakdown (Table 3) shows that the discrimination was not uniform across the three classes, and the pattern is clinically informative. The neuroendocrine class, despite being the smallest (test support ≈24 patients), was the most cleanly separated, reaching a per-class AUROC of 0.980 and a specificity of 0.984. The model almost never mistook a non-NEN patient for an NEN patient. NSCLC was recovered with the highest sensitivity (0.932) and the highest F1 (0.924). The Control class was the hardest, with the lowest per-class F1 (0.854) and recall (0.835), reflecting the deliberately heterogeneous composition of this group, which contained not only healthy individuals but also patients with benign and inflammatory pulmonary conditions whose plasma profiles partially overlap those of early malignancy. Figure 1 summarizes these results using receiver operating characteristic curves, precision–recall curves, and a confusion matrix.

As a sensitivity analysis, the three-class task was also evaluated under a strict inductive setting in which validation and test patient nodes and their incident edges were excluded from the training graph. Across ten random seeds, the inductive analysis achieved an accuracy of 0.782±0.204, a macro-F1 of 0.733±0.207, an AUROC of 0.947±0.031, and an AUPRC of 0.922±0.051. Thus, strong mean threshold-independent discrimination was retained for previously unseen patients, although threshold-dependent performance showed greater variability across splits.

### 2.3. Collapsed Cancer-Versus-Control Classification

**Figure 2:**
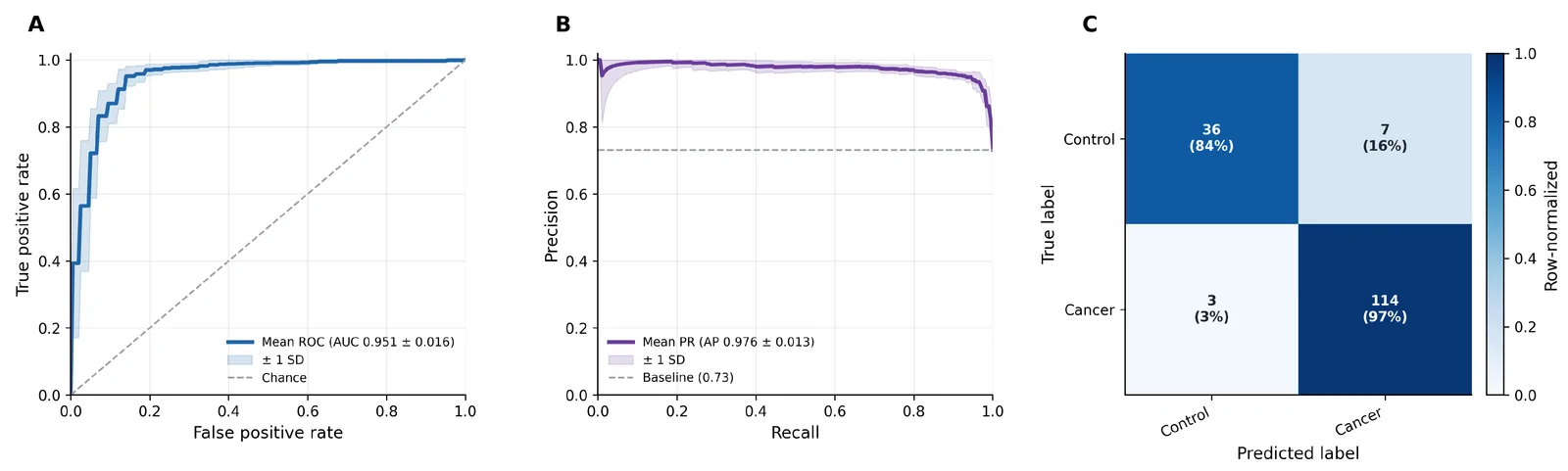
Stage 1 cancer-versus-control classification performance, with malignancy score P(NSCLC)+P(NEN). (**A**) Receiver operating characteristic curve for cancer-versus-control classification. (**B**) Precision–recall curve. (**C**) Row-normalized confusion matrix for the best-performing seed. The classification achieves high sensitivity and average precision, with residual error concentrated in specificity, consistent with benign and inflammatory pulmonary conditions in the heterogeneous Control group.

The three-class posteriors were collapsed into a single malignancy score, P(malignant)=P(NSCLC)+P(NEN), and evaluated as a binary cancer-versus-control classification (Table 4). This classification achieved an accuracy of 0.925±0.016 and an F1 of 0.949±0.010, with a sensitivity of 0.960±0.011 and an AUPRC of 0.976±0.014. The model recovered the large majority of malignancies, with the residual error concentrated in specificity (0.830±0.054), consistent with the benign-but-abnormal pulmonary conditions in the Control group being the main source of false positives (Figure 2).

### 2.4. NSCLC-Versus-NEN Subtyping

**Figure 3:**
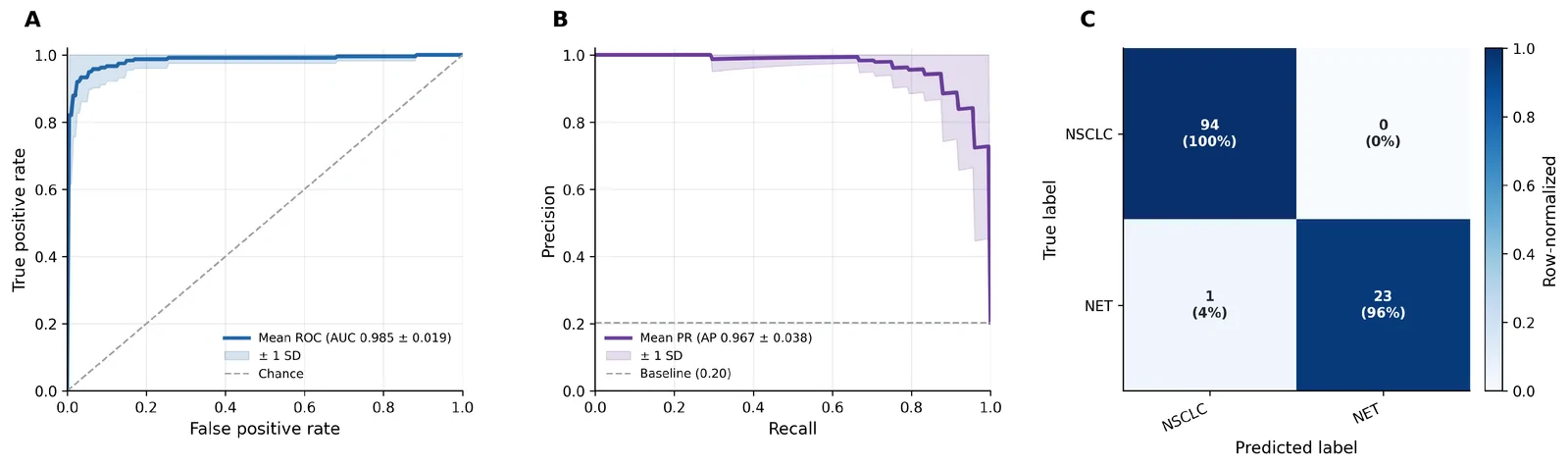
Stage 2 NSCLC-versus-NEN subtyping performance on the 586 cancer patients. (**A**) Receiver operating characteristic curve for NSCLC-versus-NEN classification. (**B**) Precision–recall curve. (**C**) Row-normalized confusion matrix for the best-performing seed. The neuroendocrine metabolic signature is highly separable from conventional NSCLC, yielding the strongest threshold-independent discrimination of any task in the study (AUROC 0.985±0.020).

In the separate cancer-only Stage 2 analysis, the classifier, trained on the 586 cancer patients only, separated pulmonary neuroendocrine neoplasms from NSCLC with an accuracy of 0.960±0.026 and an AUROC of 0.985±0.020 (Table 5). This represented the strongest discrimination among the study tasks in terms of threshold-independent metrics, with a Matthews correlation coefficient of 0.876±0.081 and a specificity of 0.983±0.019 for the NEN-positive call. The binary F1 (0.898±0.065) and its wider confidence interval reflect the small number of NEN patients available for the held-out evaluation. Within the cancer-only cohort, the neuroendocrine metabolic signature was highly separable from that of conventional NSCLC (Figure 3).

To test whether NSCLC-versus-NEN discrimination depended on the cohort-derived Class N prior, we performed a matched ablation analysis using the same ten random seeds. Within this matched rerun, the full CDR-GNN achieved an AUROC of 0.980±0.023. Removal of all 11 Class N annotations, while retaining the corresponding metabolite concentrations as measured inputs, resulted in an AUROC of 0.980±0.023, an AUPRC of 0.962±0.037, an F1 score of 0.909±0.058, and an accuracy of 0.964±0.022. The mean paired full-minus-ablated AUROC difference was −0.0001±0.0006, indicating that NSCLC-versus-NEN discrimination in the matched analysis did not depend on explicit encoding of the cohort-derived Class N panel.

The NSCLC-versus-NEN task was also evaluated under the inductive protocol. The accuracy was 0.853±0.239, the F1 was 0.677±0.370, the AUROC was 0.942±0.128, and the AUPRC was 0.901±0.210. Mean threshold-independent discrimination therefore remained high when test patients were completely absent during training, although greater between-split variability was observed than in the primary transductive analysis.

### 2.5. Comparator and Ablation Analysis

The comparator analysis (Table 6) showed that discrimination was driven primarily by metabolomic measurements. For the three-class task, the clinical-only elastic-net model achieved an AUROC of 0.700±0.026, compared with 0.958±0.016 for metabolites alone and 0.962±0.018 for the combined feature set. For NSCLC-versus-NEN classification, the corresponding AUROCs were 0.620±0.028, 0.987±0.012, and 0.984±0.014, respectively.

Paired bootstrap comparisons (Table 7) further showed no measurable improvement from the CDR prior over the otherwise matched concentration-weighted GNN. For the three-class task, the concentration-weighted GNN without CDR differed from the full CDR-GNN by ΔAUROC =0.0004 (95% CI, −0.0020 to 0.0027). For NSCLC-versus-NEN, the corresponding difference was ΔAUROC =0.0016 (95% CI, −0.0006 to 0.0056), where positive values indicate the higher performance of the comparator. Both confidence intervals included zero. The standard unweighted GNN showed a slightly lower AUROC than the full CDR-GNN for NSCLC-versus-NEN (ΔAUROC =−0.0052, 95% CI −0.0092 to −0.0018), although its paired F1 and AUPRC differences included zero. These analyses do not support a measurable predictive advantage of the CDR weighting over the concentration-weighted graph formulation. The ablation results therefore refine the interpretation of the proposed framework. The present data do not demonstrate that CDR weighting improves predictive discrimination over concentration-weighted or conventional metabolite-based models. The contribution of the CDR component lies in providing an explicit biologically motivated structure for directional metabolite and pathway-level interpretation. Predictive performance and biological interpretability should therefore be considered separate aspects of the framework.

Removing metabolite concentrations from the patient node representation while retaining them only through patient–metabolite edges substantially reduced performance, with AUROC decreasing to 0.685±0.026 for the three-class task and 0.589±0.059 for NSCLC-versus-NEN. This indicates that the direct patient-level metabolite representation is important to the current architecture.

### 2.6. Feature Attribution

**Figure 4:**
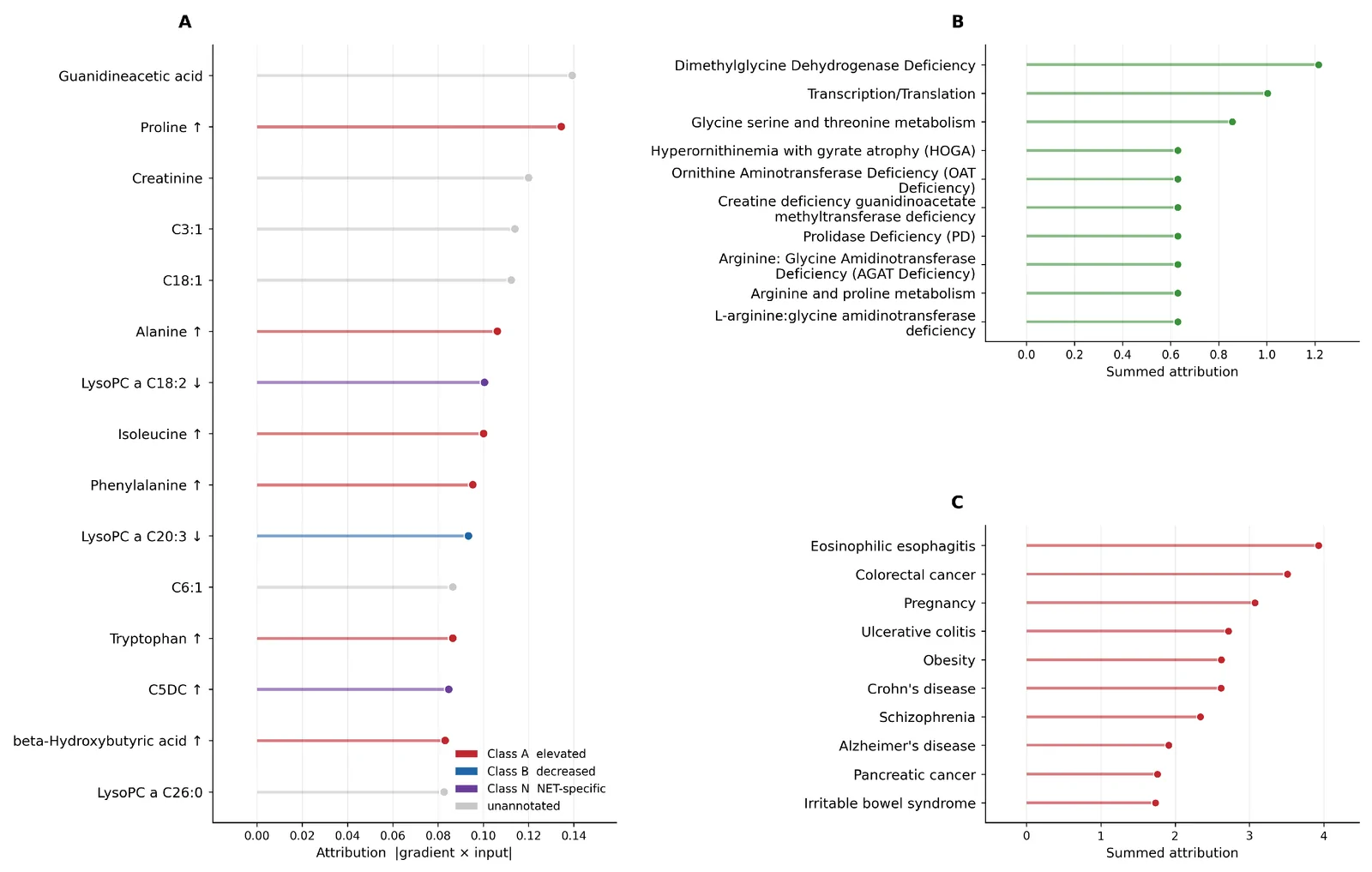
Gradient-times-input attribution for the three-class cancer signal. (**A**) Top metabolite attributions, colored by CDR class. Upward and downward arrows indicate annotated metabolites expected to be elevated and decreased, respectively. (**B**) Summed attribution of Human Metabolome Database (HMDB) pathway/disease labels. (**C**) Top HMDB disease-label attributions derived from metabolite–disease associations. HMDB labels are interpreted according to their underlying biochemistry.

**Figure 5:**
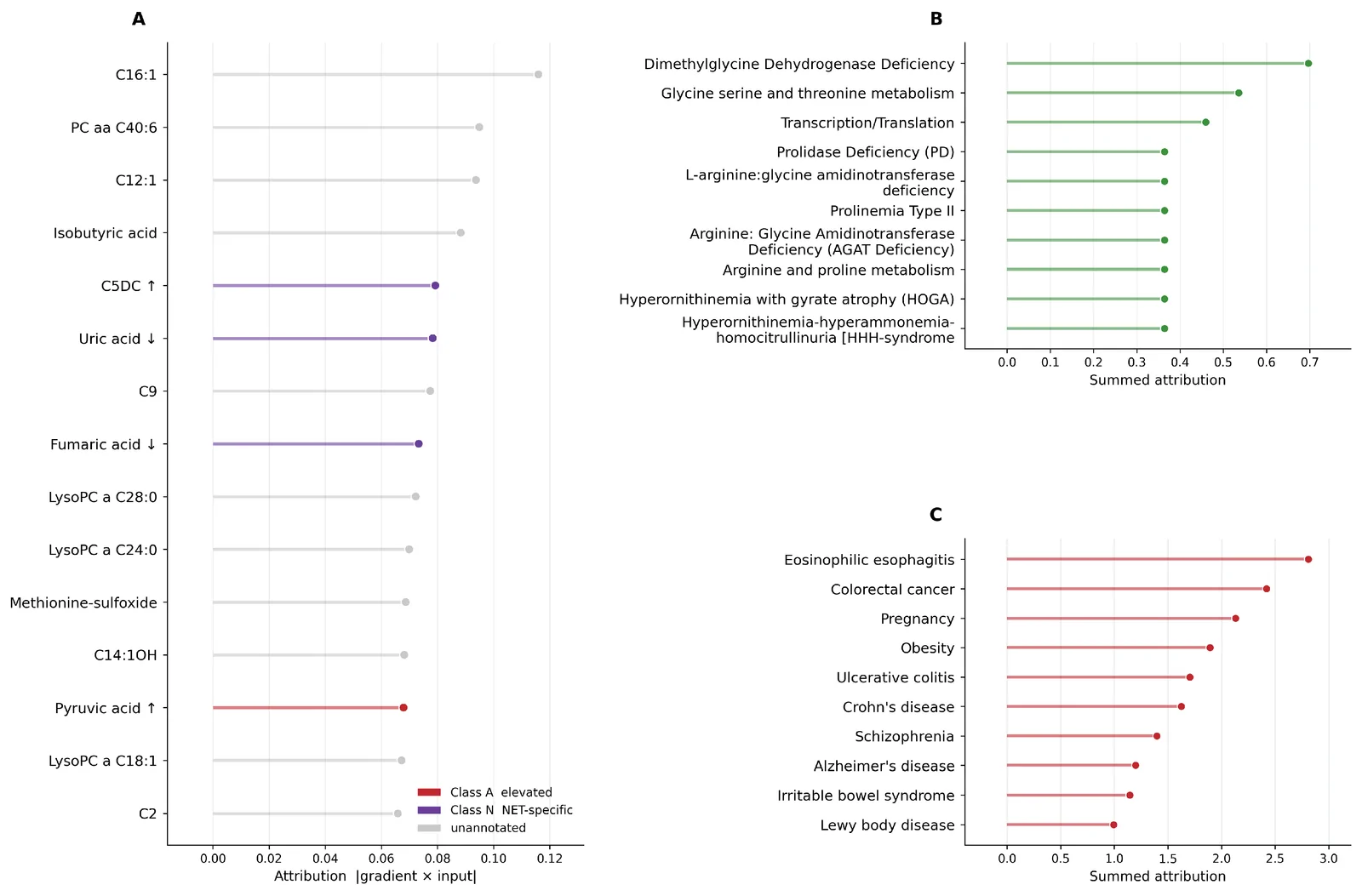
Gradient-times-input attribution for NSCLC-versus-NEN subtyping task. (**A**) Top metabolites, colored by CDR class. The three highest-attribution annotated features are all Class N neuroendocrine markers, C5DC (elevated), uric acid (decreased), and fumaric acid (decreased), with the remainder of the leaders unannotated. (**B**) Pathway/disease labels, led by Dimethylglycine Dehydrogenase Deficiency, glycine, serine and threonine metabolism, and Transcription/Translation, followed by a tight proline and ornithine–arginine cluster (≈0.36). (**C**) Top HMDB disease labels, interpreted by their underlying biochemistry.

The gradient-times-input attribution [14] on the held-out test patients of the best seed was aggregated to the metabolite and pathway nodes for both the three-class cancer signal (NSCLC + NEN) and the NSCLC-versus-NEN subtyping task. At the pathway level (Figure 4B), the single strongest contributor to the three-class cancer signal was the Dimethylglycine Dehydrogenase Deficiency pathway (summed attribution 1.21), followed by the broad Transcription/Translation pathway (1.00) and glycine, serine and threonine metabolism (0.86). A cluster of twelve lower-ranked pathways carried nearly identical attribution (≈0.63) and comprised proline-related labels (Hyperprolinemia Types I and II, Prolinemia Type II, Prolidase Deficiency, and arginine and proline metabolism) together with ornithine–arginine–creatine labels (the two Hyperornithinemia pathways, Ornithine Aminotransferase Deficiency, the two Amidinotransferase pathways, and the Guanidinoacetate Methyltransferase pathways). Because these twelve values are indistinguishable, we treat them as a single cluster. The same dimethylglycine and glycine–serine signal led to the NSCLC-versus-NEN subtyping attribution (Figure 4 and Figure 5), with glycine, serine and threonine metabolism appearing explicitly as the second-ranked pathways.

At the individual-metabolite level, the named annotated metabolites with the highest attribution were proline (an elevated, Class A feature), followed by the amino acids alanine, isoleucine, and phenylalanine and the decreased Class N feature LysoPC a C18:2 for the three-class cancer signal, and the neuroendocrine class N markers C5DC (elevated), uric acid (decreased), and fumaric acid (decreased) for the NSCLC-versus-NEN task. A substantial portion of the highest per-metabolite attribution, however, fell on metabolites that carried no CDR annotation, indicating that the network draws on signal beyond the curated directional prior. The interpretation we develop below therefore rests on the pathway-level aggregation, which is more robust to individual unannotated features, with the named CDR metabolites read as corroborating anchors.

We trained a heterogeneous graph neural network that encodes plasma metabolomics together with a curated Cell Danger Response (CDR) directional prior, and showed that it discriminates Control, NSCLC, and pulmonary neuroendocrine neoplasms with a macro-AUROC of 0.962, classifies cancer versus control with a sensitivity of 0.960, and distinguishes NSCLC from NEN with an AUROC of 0.985.

The present CDR-GNN extends the previously published M-GNN framework [13] while retaining the same core GraphSAGE and graph-attention encoder. M-GNN was developed for binary lung cancer-versus-control classification using metabolite concentrations together with HMDB-derived disease and pathway relationships. CDR-GNN extends this framework by adding CDR class annotations and direction-aware patient–metabolite weighting, and by expanding the prediction task to Control, NSCLC, and NEN classification with a separate NSCLC-versus-NEN analysis.

The previously published M-GNN achieved an accuracy of 0.885±0.038, an F1 score of 0.922±0.028, an AUROC of 0.923±0.026, and an AUPRC of 0.962±0.016. The corresponding cancer-versus-control values in the present CDR-GNN were 0.925±0.016, 0.949±0.010, 0.951±0.017, and 0.976±0.014, respectively. These differences should be interpreted descriptively because the present framework also uses an expanded metabolite set and a multiclass training objective.

The baseline analysis also identified clinical differences between the diagnostic groups, particularly in smoking history and cumulative smoking exposure. Ever-smoking status showed an SMD of 1.09 between Control and NSCLC and 0.62 between Control and NEN, while pack-year exposure showed corresponding SMDs of 0.62 and 0.56. Age also differed between Control and NSCLC (SMD =0.46). These imbalances are important because demographic and smoking characteristics could contribute to apparent diagnostic discrimination. However, clinical variables alone provided substantially weaker discrimination than metabolite concentrations: clinical-only AUROC was 0.700±0.026 for the three-class task and 0.620±0.028 for NSCLC-versus-NEN, compared with 0.958±0.016 and 0.987±0.012, respectively, for metabolites alone. Thus, measured clinical variables alone do not explain the observed performance, although residual confounding cannot be excluded in this retrospective cohort.

The pooled NEN class combines histologically and clinically distinct entities. Carcinoid tumors are generally better differentiated and less aggressive than the high-grade neuroendocrine carcinomas SCLC and LCNEC, and these entities differ substantially in proliferation, prognosis, and clinical management [7]. They were pooled because the present objective was to identify a broader pulmonary neuroendocrine metabolic phenotype relative to conventional NSCLC. Pulmonary NENs share neuroendocrine differentiation and exhibit common pathway-level metabolic perturbations while also showing subtype-specific metabolite differences [6]. Accordingly, the pooled results should not be interpreted as evidence that carcinoid, SCLC, and LCNEC are metabolically equivalent. Larger subtype-stratified cohorts will be required to establish histology-specific performance and generalizability.

Aggregating the attribution along the pathway edges produced a small and biochemically coherent set of programs, although the labels require cautious interpretation because the Human Metabolome Database catalogs many metabolites under the rare inborn errors of metabolism in which they are classically abnormal [15]. We therefore read each pathway by its underlying biochemistry (Figure 4B and Figure 5B).

The strongest single contributor, Dimethylglycine Dehydrogenase Deficiency, together with glycine, serine and threonine metabolism, maps to one-carbon and glycine–serine metabolism, a program that supplies proliferating cells with nucleotides and methyl groups and represents an established cancer dependency [16]. The accompanying creatine and guanidinoacetate labels are also consistent with this interpretation, as the methylation of guanidinoacetate to creatine is the largest single consumer of S-adenosylmethionine-derived methyl groups [17]. The fact that a one-carbon and glycine–serine signature emerged as the leading discriminator is biologically reasonable, because serine–glycine one-carbon flux supports nucleotide and glutathione synthesis and is repeatedly implicated as a vulnerability in lung and other cancers [18,19].

A second cluster of pathways, comprising Hyperprolinemia Types I and II and Prolidase Deficiency, corresponds to proline and pyrroline-5-carboxylate metabolism, which supports redox balance, pyridine nucleotide maintenance, and proliferation in many tumors [20]. The two Hyperornithinemia pathways and the creatine/guanidinoacetate labels reflect the ornithine, arginine, and urea-cycle axis that cancers rewire to maximize the use of nitrogen for anabolic biosynthesis [21]. These proline and ornithine–arginine labels carried statistically indistinguishable attribution (≈0.63), so we describe them as a single proline and arginine–ornithine cluster. The remaining broad Transcription/Translation pathway denotes a generic amino acid availability category.

The named metabolite-level anchors are consistent with this pathway reading and with the known biology of the cohort. For the NSCLC-versus-NEN task, the highest-attribution annotated metabolites were all neuroendocrine class N markers identified by Boullier et al. across this cohort: the acylcarnitine C5DC (elevated), uric acid (decreased), and the tricarboxylic-acid-cycle intermediate fumaric acid (decreased) [6]. For the three-class cancer signal, the highest-attribution annotated metabolite was proline (elevated, Class A), which sits on the same proline and pyrroline-5-carboxylate axis that dominated the pathway-level cluster, with the amino acids alanine, isoleucine, and phenylalanine following.

Several metabolites receiving high attribution in the NSCLC-versus-NEN analysis were previously identified as neuroendocrine-associated metabolites in the companion cohort, including C5DC, uric acid, and fumaric acid (Figure 5A). Their recurrence in the attribution analysis should therefore be interpreted as consistency with the prior-informed representation. For the three-class cancer signal, proline was the highest-attribution annotated metabolite, followed by alanine, isoleucine, and phenylalanine (Figure 4A).

To determine whether NSCLC-versus-NEN classification performance depended on this cohort-derived information, we repeated the analysis after removing all 11 Class N annotations while retaining the corresponding metabolite concentrations as measured inputs. Performance remained essentially unchanged, with an AUROC of 0.980±0.023 for both the full and Class N-ablated models. This finding indicates that the high NSCLC-versus-NEN discrimination was not driven by explicit encoding of the previously identified Class N metabolite panel. Nevertheless, the present evaluation remains internal to this cohort, and independent external validation is required to establish the generalizability of the observed metabolomic associations.

The directional annotation used here is named for, and motivated by, the Cell Danger Response (CDR) framework of Naviaux [22,23]. The CDR describes an evolutionarily conserved metabolic state that cells enter when they encounter threat, whether infectious, chemical, or physical, in which mitochondria shift from energy production toward defense, extracellular nucleotides such as ATP act as danger signals through purinergic receptors, and the cell transits through a sequence of healing phases with characteristic bioenergetic signatures. Malignant transformation can be read, in this framework, as a CDR that fails to resolve. Tumor cells remain locked in a defensive, proliferative metabolic state with sustained glycolytic flux, altered tricarboxylic acid cycle activity, and disrupted purine and lipid handling [24].

Several of the metabolites that the attribution analysis surfaced map onto core elements of the CDR. Among them, uric acid, a leading discriminator in the NSCLC-versus-NEN task, is the terminal product of purine catabolism. Because extracellular ATP and its breakdown products are central danger signals in the CDR, perturbed purine turnover is exactly the kind of marker the framework predicts should carry information about a dysregulated danger state [22]. Another leading discriminator, fumaric acid, is a Class N feature that is decreased in the neuroendocrine group and a tricarboxylic-acid-cycle intermediate, consistent with the mitochondrial remodeling described by the CDR [23] and with the altered TCA cycle activity reported in this cohort [6]. The acylcarnitine C5DC, the highest-attribution neuroendocrine marker in this task, reports on the mitochondrial acyl-group metabolism that shifts during a sustained danger state [23]. At the pathway level, the proline and ornithine–arginine cluster and the one-carbon and glycine–serine signature that dominated the attribution are consistent with the redox-balancing, biosynthetic, and nitrogen-handling adaptations expected of a sustained, unresolved danger state.

The disease nodes in the graph are drawn from the Human Metabolome Database’s metabolite–disease associations (Figure 4C and Figure 5C). A disease label accumulates attribution when the metabolites the network relies on are the same species that are perturbed in that condition, which implies shared underlying pathways or mechanisms. Several of the highest-scoring disease nodes are interpretable on exactly these grounds. Other malignancies, including colorectal and pancreatic cancer, share the proliferative serine–glycine–one-carbon and amino acid reprogramming that defines cancer metabolism broadly [19], so their appearance indicates that the model keys on generic malignant–metabolic hallmarks. Chronic inflammatory bowel conditions, Crohn’s disease and ulcerative colitis, are associated with documented serum amino acid and energy–metabolism perturbations, including altered tryptophan handling [25], that overlap the same amino acid and one-carbon axes the attribution surfaced and the danger-state biology of the CDR. Obesity, a state of systemic metabolic stress, carries a well-characterized signature of elevated branched-chain and aromatic amino acids [26], precisely the pool from which isoleucine and phenylalanine emerged among the model’s leading features. The co-attribution of these otherwise unrelated conditions therefore reflects a small number of shared metabolite hubs, including amino acids, one-carbon intermediates, and purine end-products such as uric acid, that sit at the intersection of proliferation, inflammation, and metabolic stress, and this convergence is consistent with the model having learned biology common to cellular danger states. Nonetheless, these remain biochemical tags read through their shared pathways and are not diagnoses inferred for any participant. Summed attribution favors labels that simply have many associated metabolites, so apparent rank partly reflects catalog breadth. In addition, a single metabolite can map to several diseases, so neighboring entries are often redundant reflections of the same underlying biochemistry.

Two cautions bound this interpretation. First, Naviaux assigns bioenergetic signatures to broad metabolic phases and cell states, not to the specific named plasma metabolites of a targeted assay. The mapping from individual panel metabolites onto the CDR is therefore an interpretive bridge that we draw at the level of pathway biochemistry, not a set of quantitative directional values published in the original work. We do not claim that the edge boost prescribes per-metabolite directions. Those directions are drawn from the cancer metabolomics literature and from the companion cohort study [6], with the CDR providing the unifying conceptual rationale. Second, as emphasized above, the prior functions as an interpretable scaffold, so the value of the CDR connection lies in rendering the learned signature biologically legible and falsifiable. A natural direction for future work is to test whether CDR-phase-specific priors capture the resolution versus the non-resolution of the danger state across tumor subtypes, a hypothesis that would require independent cohorts and phase-resolved metabolic readouts to evaluate.

An additional direction for future work is the integration of plasma metabolomics with circulating cell-free DNA (cfDNA) methylation. Dong et al. demonstrated the feasibility of blood-based pan-cancer detection using cfDNA methylation markers, including HIST1H4F and CDO1 [27]. Within a graph-based framework, these molecular layers could be represented as complementary patient features or additional node and edge types.

Pulmonary neuroendocrine neoplasms can present as peripheral nodules that are difficult to distinguish from NSCLC on low-dose computed tomography, yet their treatment pathways diverge sharply, from somatostatin-analog and peptide-receptor approaches for well-differentiated carcinoids to platinum-etoposide regimens for high-grade neuroendocrine carcinomas, versus the surgical, targeted, and immunotherapeutic pathways of NSCLC [28,29]. A non-invasive plasma assay that first flags malignancy at high sensitivity and then identifies the neuroendocrine metabolic signature at high specificity could help direct biopsy strategy and preliminary management while tissue confirmation is pursued. The high discrimination (AUROC 0.985) is encouraging in this regard, although the modest number of NEN patients available for held-out evaluation means the subtyping operating point should be confirmed in a larger, prospectively collected cohort before any clinical inference is drawn.

## 3. Materials and Methods

### 3.1. Study Population and Plasma Collection

This investigation used a retrospective case–control design based on an expanded cohort of 800 plasma samples obtained through the Institut Universitaire de Cardiologie et de Pneumologie de Québec–Université Laval (IUCPQ-UL) biobank. The cohort comprised 586 lung cancer cases and 214 non-cancer Controls. The cancer group included 466 patients with non-small-cell lung cancer and 120 patients with pulmonary neuroendocrine neoplasms (NENs). Among the NEN cases, 50 were classified as carcinoid tumors, 40 as small-cell lung carcinomas (SCLCs), and 30 as large-cell neuroendocrine carcinomas (LCNECs), representing a spectrum from less aggressive carcinoid tumors to high-grade pulmonary neuroendocrine carcinomas. The Control group was itself heterogeneous, incorporating 80 healthy individuals with no documented pulmonary pathology alongside 134 patients presenting with non-cancerous lung conditions including hamartoma, apical cap, asthma, bronchiectasis, chronic obstructive pulmonary disease (COPD), and COVID-19 pneumonia. This broadening of the Control class, relative to cohorts that recruit only healthy volunteers, increases the clinical applicability of the resulting classifier to a realistic primary care or respiratory clinic setting where incidental pulmonary findings must be distinguished from malignancy across a background of benign and inflammatory lung disease. The three pulmonary neuroendocrine histologies were analyzed as a single NEN class because the primary objective was to distinguish a broader pulmonary neuroendocrine phenotype from conventional NSCLC. This aggregation was not intended to imply biological equivalence among carcinoid tumors, SCLC, and LCNEC.

### 3.2. Cohort Characteristics and Clinical Balance

The largest between-group imbalances involved smoking history and cumulative smoking exposure. Ever-smoking prevalence differed substantially between Control and NSCLC (SMD =1.09) and between Control and NEN (SMD =0.62). Pack-year exposure showed corresponding SMDs of 0.62 and 0.56. Additional imbalance was observed for age between Control and NSCLC (SMD =0.46) and between NSCLC and NEN (SMD =0.37). These imbalances motivated explicit clinical-only, metabolomics-only, and combined comparator analyses.

All samples underwent fully quantitative targeted mass spectrometry covering 166 metabolites. Profiling was carried out on an Agilent 1100 HPLC system (Agilent Technologies, Santa Clara, CA, USA) coupled to an AB Sciex 4000 QTrap^®^ tandem mass spectrometer (Applied Biosystems/MDS Analytical Technologies, Foster City, CA, USA). Plasma was thawed on ice, vortex-mixed, and centrifuged at 18,000× *g*. For the amino acid, biogenic amine, acylcarnitine, and lipid panels, a 10 µL aliquot of each sample was applied to a filter insert in a 96-well plate and evaporated to dryness under nitrogen. Metabolites were then derivatized with phenyl isothiocyanate (PITC) and eluted with methanol containing 5 mM ammonium acetate, with the eluate recovered by centrifugation of the stacked-plate assembly to enable targeted identification and quantification. These extracts were diluted as appropriate prior to mass spectrometric acquisition. Organic acids were processed separately. A 50 µL aliquot of plasma was combined with an internal-standard mixture and ice-cold methanol and left to precipitate protein overnight at −20 °C, after which the supernatant was derivatized with 3-nitrophenylhydrazine (3-NPH) and accompanying reagents before injection into the HPLC-MS/MS system. Isotope-labeled internal standards were incorporated throughout to support accurate quantification, and the assay spanned amino acids, organic acids, biogenic amines, acylcarnitines, glycerophospholipids, sphingolipids, and sugars.

Metabolites for which more than 30% of samples yielded missing values were excluded from further analysis, yielding 150 quantifiable metabolites for computational modeling. Values falling below the instrument limit of detection (LOD) were replaced during initial data assembly with published LOD values from the same assay, representing a conservative imputation strategy that preserves relative ordering at the lower end of the quantification range. Residual missingness within the modeling pipeline was addressed using k-nearest neighbor imputation (k=2).

### 3.3. Cell Danger Response Annotation

To encode prior knowledge about the directional behavior of metabolites in malignancy into the graph, each metabolite that could be matched to the assay was assigned a Cell Danger Response (CDR) class together with a relevance score and an expected direction of change. Thirty-seven metabolites were annotated and received boosted edges. Table 8 summarizes the lung-cancer directional metabolite priors used to construct CDR-informed patient–metabolite edge weights. Metabolites in Class A were expected to be elevated in lung cancer, so positive standardized deviations increased the corresponding edge abnormality score. Metabolites in Class B were expected to be decreased in lung cancer, so negative standardized deviations increased the edge abnormality score. All listed metabolites received the same edge boost of 1.5, ensuring that the prior biological evidence affected edge weighting uniformly while preserving the direction-specific interpretation of metabolite abnormality.

The directional assignments were grounded in the cancer metabolic-reprogramming literature and in established metabolic stress-response biology [22,23]. Class N (n=11) encoded neuroendocrine-specific metabolites identified by Boullier et al. in the companion study [6].

The annotation entered the model in two ways. Metabolite nodes received a five-dimensional CDR feature vector consisting of a binary annotation indicator and a four-dimensional one-hot encoding of Classes A, B, C, and N. For unannotated metabolites, all five CDR features were set to zero. Thus, unannotated metabolites did not constitute an additional CDR class. These features were appended to the metabolite node feature vector (Section 3.4). A single uniform multiplicative boost was then applied to the patient-to-metabolite edge of every annotated metabolite irrespective of class. We adopted one shared boost deliberately so that the network did not encode unvalidated class-specific importance differences that the data could not justify. The expected direction of change instead determined how each patient’s standardized metabolite value contributed to the direction-aware edge abnormality score (Section 3.4).

From the biological perspective, pulmonary NENs represent a distinct cellular lineage that arises from APUD (amine precursor uptake and decarboxylation) neuroendocrine cells resident in the bronchial epithelium and not from the type II pneumocytes or basal airway cells that give rise to adenocarcinoma and squamous cell carcinoma, respectively [45,46,47]. This distinct cellular origin confers distinct metabolic programming. The amine-secreting neuroendocrine phenotype is reflected in circulating metabolites such as the serotonin catabolite 5-hydroxyindoleacetic acid, and Boullier et al. additionally reported alterations in fatty acid biosynthesis and beta-oxidation, the Warburg effect, the citric acid cycle, and lysophosphatidylcholine levels across pulmonary neuroendocrine neoplasms; these are profiles that distinguish them in plasma from both healthy individuals and NSCLC [6].

NENs are notoriously difficult to distinguish from other malignancies by low-dose CT imaging because they can present as peripheral nodules indistinguishable from NSCLC at the early nodule stage [48]. A non-invasive liquid biopsy approach that can flag the neuroendocrine metabolic signature in plasma therefore has potential clinical utility for guiding biopsy approach and preliminary treatment planning.

The three classes assigned were as follows: Control (y=0, n=214), encompassing all non-cancer participants, including the heterogeneous non-cancerous lung disease group; NSCLC (y=1, n=466), comprising all NSCLC cases; NEN (y=2, n=120), comprising all NEN cases, regardless of histological subtype. The clinical significance of separating the two cancer classes lies in the divergence of treatment pathways. Pulmonary NENs exhibit neuroendocrine features related to amine handling and peptide secretion, as well as plasma metabolomic alterations involving lipid remodeling, Warburg-related metabolism, and tricarboxylic acid cycle activity [6,49,50]. These subtype-associated metabolic differences support the use of plasma metabolomic profiles for discriminating pulmonary NENs from Controls and from conventional NSCLC in the studied cohort.

### 3.4. Heterogeneous Graph Construction

**Figure 6:**
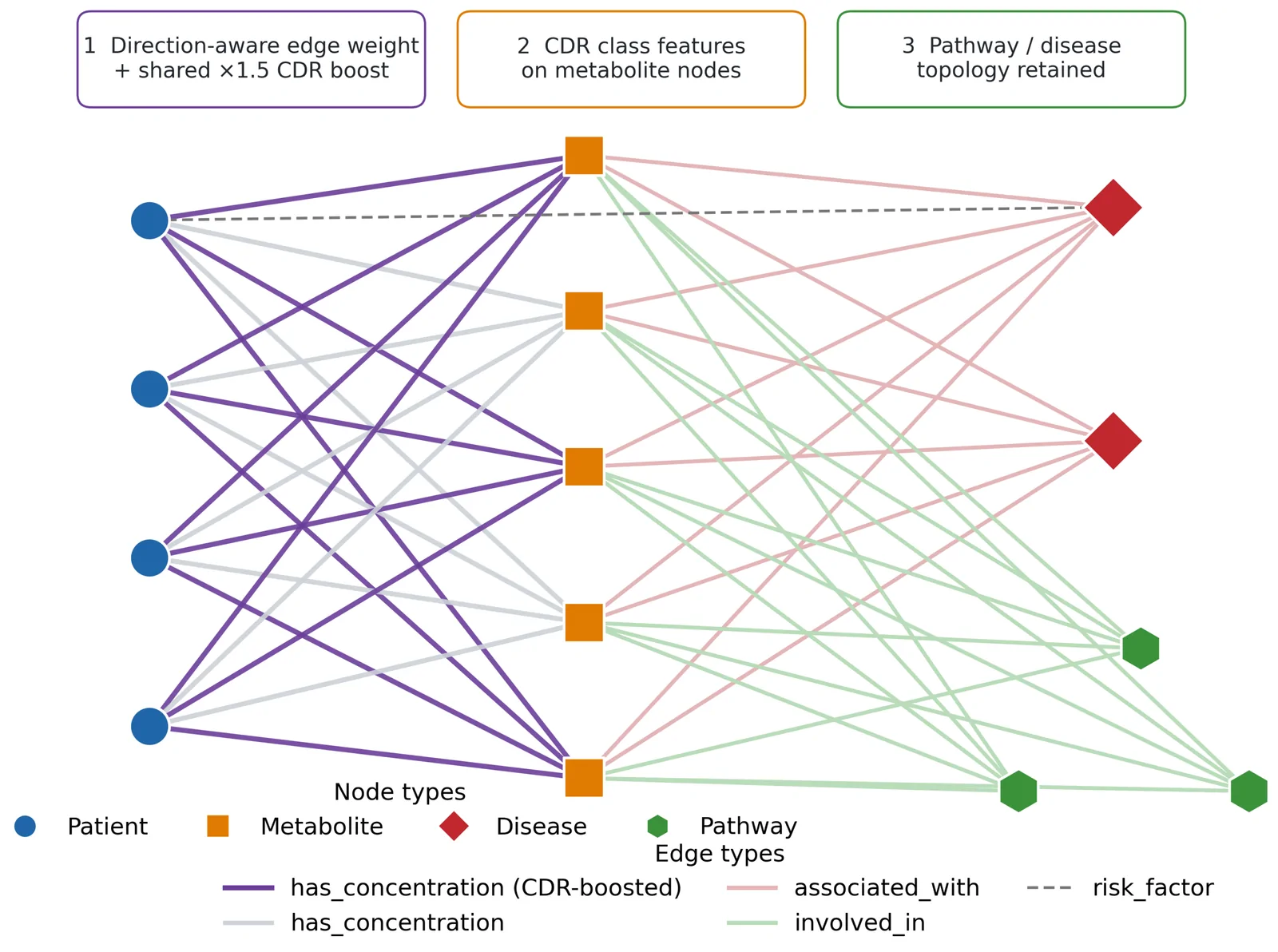
Schematic of the heterogeneous attributed graph. Four node types are encoded: patients (circles), metabolites (squares), diseases (diamonds), and metabolic pathways (hexagons). Four edge relations connect them: *has_concentration* edges link patients to measured metabolites and are direction-aware and carry the shared ×1.5 CDR boost on annotated metabolites (callout 1), *associated_with* edges link metabolites to HMDB disease nodes, *involved_in* edges link metabolites to pathway nodes, and *risk_factor* edges (dashed) connect patients who are current or former smokers to the lung-cancer disease node. The CDR class and relevance features are attached to the metabolite nodes (callout 2), while the disease and pathway topology is retained unchanged from the underlying knowledge graph (callout 3).

This work encodes patient metabolomics data as a heterogeneous attributed graph (Figure 6) G=(V,E), constructed using the NetworkX library [51] and converted to a PyTorch Geometric [52] data object using PyTorch (version 2.7.0) and past smoking history, five continuous clinical measurements (age, height, weight, body mass index, and cumulative pack-year exposure), and the metabolite concentrations. Metabolite nodes received an eight-dimensional feature vector comprising the assay normal-range lower bound, upper bound, and population mean, a binary CDR annotation indicator, and a four-dimensional one-hot encoding of CDR Classes A, B, C, and N. Unannotated metabolites received zero values for the annotation indicator and all four class indicators. Disease nodes, added for each disease associated with one or more metabolites in the HMDB disease–metabolite association database, and pathway nodes, added for each metabolic pathway, carried zero feature vectors and served as structural anchors that allow information to propagate between metabolites sharing a disease association or pathway, even when those metabolites are not directly connected. Patient, metabolite, disease, and pathway node features were projected into a common hidden dimension using node-type-specific input projections before message passing.

Four edge types were defined and symmetrized for undirected message passing. The primary type, has_concentration, connected each patient to each metabolite with a measured value, and its weight encoded both how abnormal the value was and whether the abnormality was in the biologically expected direction. For metabolite *m* in patient *p*, $z_{pm}$ denotes the training-standardized concentration. The direction-aware abnormality $a(z_{pm},m)$ was defined as

(1) $$a\left(z_{pm},m\right)=\left\{\begin{array}{cc} \max(z_{pm},0) & \mathrm{if}\;m\;\mathrm{is}\;\mathrm{annotated}\;\mathrm{as}\;\mathrm{elevated},\\ \max(-z_{pm},0) & \mathrm{if}\;m\;\mathrm{is}\;\mathrm{annotated}\;\mathrm{as}\;\mathrm{decreased},\\ \left|z_{pm}\right| & \mathrm{if}\;m\;\mathrm{is}\;\mathrm{unannotated}. \end{array}\right.$$

Thus, for an annotated metabolite, the edge is strengthened only when the patient’s metabolite concentration deviates in the expected direction. The patient–metabolite edge weight w(p,m) was defined as

(2) $$w\left(p,m\right)=\log\;\left(1+a(z_{pm},m)\right)\;\varphi \left(m\right),\;\varphi \left(m\right)=\left\{\begin{array}{cc} 1.5 & \mathrm{if}\;m\;\mathrm{is}\;\mathrm{CDR}\text{-}\mathrm{annotated},\\ 1.0 & \mathrm{otherwise}. \end{array}\right.$$

For an unannotated metabolite, which carries no directional prior, any departure from the population mean strengthens the edge through its absolute standardized deviation. The logarithm compresses extreme deviations and φ is the single uniform CDR boost of the preceding section, applied identically to every annotated metabolite. The associated_with edges linked each metabolite to its HMDB disease nodes with weight 1.0, or 2.0 for lung cancer associations. The involved_in edges linked each metabolite to its metabolic pathway nodes with weight 1.0. The risk_factor edges connected patient nodes representing current or former smokers to the lung cancer disease node with a weight of 0.8, encoding the established link between tobacco exposure and NSCLC risk. Each edge carried its scalar weight together with a one-hot encoding of its relation type, and the symmetrized graph was dominated by the patient-to-metabolite concentration edges.

During each message-passing step, the scalar edge weight was incorporated into the neighborhood aggregation according to

(3) $$h_{v}^{\left(\ell +1\right)}=h_{v}^{(\ell +1),\;\mathrm{SAGE}}+\frac{\sum_{u\in N(v)}\sigma \;\left(w_{uv}\gamma \right)h_{u}^{\left(\ell \right)}}{\max\;\left(\deg(v),1\right)},$$

where $h_{v}^{(\ell +1),\;\mathrm{SAGE}}$ is the standard SAGEConv output for node *v* at layer ℓ+1, $w_{uv}$ is the edge weight defined in Equation (2), and γ is a learned scalar initialized at 0.5 and passed through a sigmoid to bound the influence of the edge weight. The denominator normalizes the weighted neighborhood contribution by node degree to prevent high-degree patient nodes from dominating the gradient signal. This formulation extends the weighted adjacency adjustment of the original M-GNN [13] to the CDR three-class classification task introduced here.

### 3.5. Model Architecture and Training

The classifier was the M-GNN encoder of Vaida et al. [13], a three-layer message-passing network. An initial SAGEConv layer projects the input to a hidden width of 128 and applies the weighted adjacency adjustment of Equation (3), followed by batch normalization, an ELU activation, and dropout with probability 0.3. A multi-head graph-attention layer with eight attention heads, receiving the edge weights as scalar attention guidance, is followed by a second SAGEConv layer that again applies the weighted adjacency adjustment. A two-layer classifier head reduces the 128-dimensional representation through a 32-dimensional bottleneck to the class logits. The edge-influence scalar, γ, of Equation (3) is the only learned edge parameter.

For each of ten pre-specified random seeds, the 800 patients were partitioned at the patient level into training (60%), validation (20%), and test (20%) sets by stratified sampling that preserved class proportions across all three splits. Splitting preceded any data-dependent preprocessing. Within each seed, k-nearest-neighbor imputation (k=2) and standard scaling were fit on the training partition only. The substantial training-set class imbalance (NSCLC n≈280, Control n≈128, and neuroendocrine n≈72) was addressed using the Synthetic Minority Oversampling Technique, applied to the training partition only. Synthetic patient nodes entered the graph as ordinary patient nodes and contributed to gradient computation during training but were excluded from validation and test evaluation so that all reported metrics reflect genuine biological samples only and no synthetic sample ever appears in a held-out set.

The primary analysis used a transductive node-classification setting. Training, validation, and test labels were separated by masks, while the real patient nodes and their features remained present in the shared graph during message passing. Test labels did not contribute to optimization, model selection, imputation, normalization, or oversampling.

To assess generalization to previously unseen patients, we additionally performed a strict inductive sensitivity analysis. For each random seed, training, validation, and test graphs were constructed separately after patient splitting. Test patient nodes and their incident edges were therefore completely absent during model training. The biological metabolite, pathway, and disease structure was retained across graphs, while all data-dependent preprocessing remained fit on the training partition only.

The model was optimized using AdamW with an initial learning rate of $7\times 10^{-5}$ and weight decay of $1\times 10^{-11}$. The learning rate was reduced by a factor of 0.5 when validation loss failed to improve, and the gradient norm was clipped to a maximum value of 1.0 at each update. Cross-entropy loss was computed with inverse-frequency class weights to compensate for residual class imbalance, and label smoothing of ε=0.01 was applied. Model selection was based on macro-averaged F1 on the validation set, with early stopping triggered after 300 epochs without improvement and a maximum budget of 1500 epochs. The test partition of each seed was evaluated only once using the checkpoint selected on the validation set. Performance statistics are reported as mean ± standard deviation across the ten fixed random seeds.

**Figure 7:**
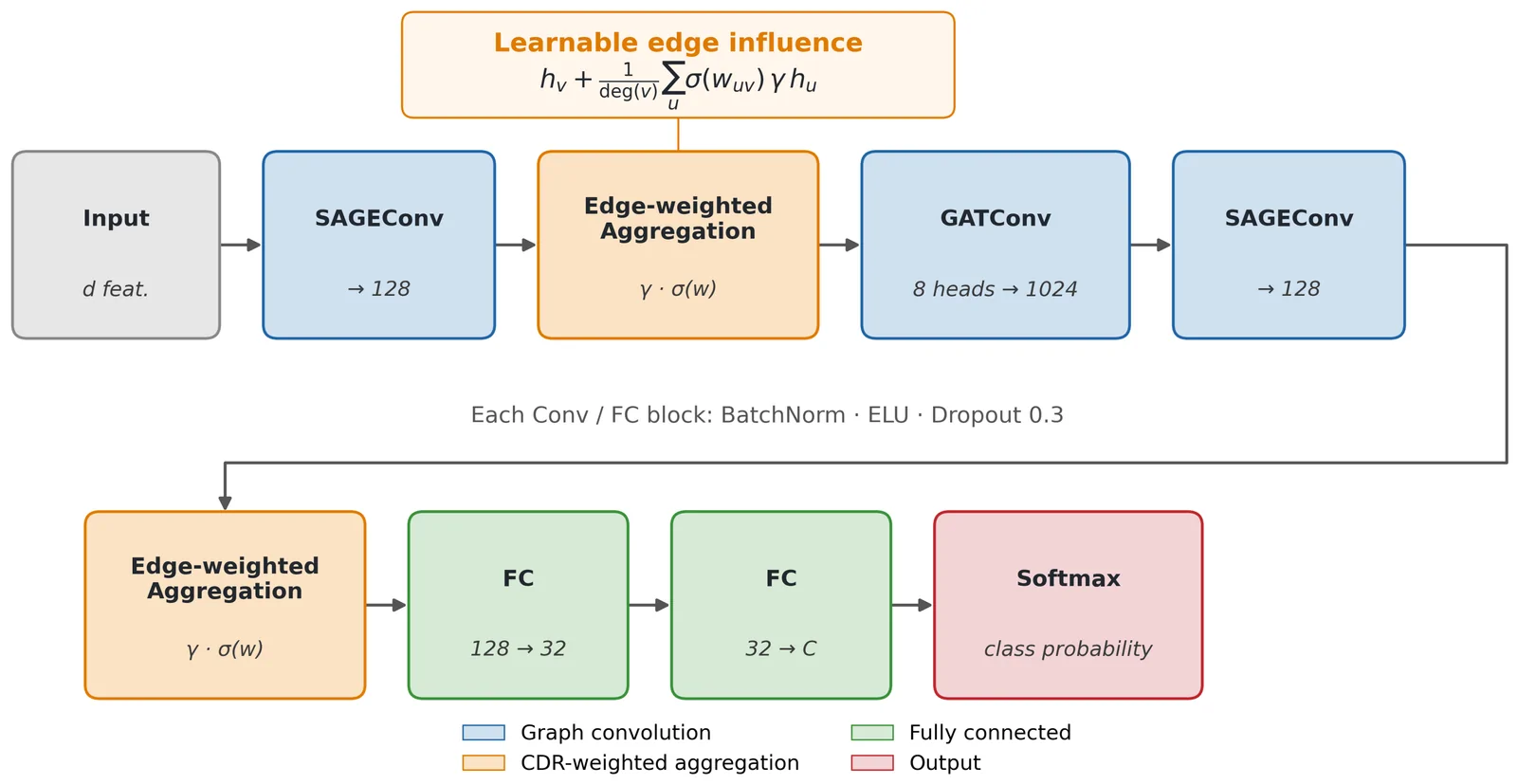
The M-GNN encoder. The 159-dimensional input is passed through a SAGEConv layer (→128), a CDR edge-weighted aggregation block implementing Equation (3), an eight-head graph-attention layer (8×128=1024), a second SAGEConv layer (→128) with a further edge-weighted aggregation block, and a two-layer fully connected head (128→32→C) producing the class posteriors via softmax. The orange callout shows the learnable edge-influence aggregation, in which the single scalar γ gates the CDR-weighted neighbor sum. Each convolutional and fully connected block applies batch normalization, an ELU activation, and dropout with probability 0.3.

The three-class posteriors were collapsed into a scalar malignancy score, P(malignant)=P(NSCLC)+P(NEN), which was evaluated for cancer-versus-control classification. A binary classifier, using the same encoder architecture but trained independently from scratch on the 586 cancer patients only (NSCLC n=466, NEN n=120) with training-partition oversampling, discriminated NEN from NSCLC. Both analyses followed the same patient-level split, training-only preprocessing, and ten-seed transductive evaluation protocol. The full encoder is summarized in Figure 7.

Biological interpretation used gradient-times-input attribution [14] on the held-out test patients of the best seed. For the target class or classes, the product of each input feature and its gradient was taken in absolute value and averaged over the test patients, then aggregated at the metabolite, pathway, and disease nodes through the corresponding edges to yield ranked attributions.

To determine the relative contribution of clinical variables, metabolomic measurements, graph structure, concentration weighting, and the CDR prior, we performed a matched comparator and ablation analysis using the same ten patient-level random seeds and train–validation–test partitions as the primary analysis. Conventional tabular comparators included clinical-only, metabolites-only, and combined clinical-plus-metabolites elastic-net logistic regression, as well as random forest and multilayer perceptron classifiers. Model hyperparameters were selected using validation-set AUROC only.

Graph ablations included a standard unweighted GNN, a concentration-weighted GNN without CDR information, a uniform CDR-boost model without directional weighting, a directional-prior model without the CDR boost, a CDR node-prior-only model, the full CDR-GNN, and a full CDR-GNN in which metabolite concentrations were removed from patient node features and retained only on patient–metabolite edges. All data-dependent preprocessing and SMOTE were restricted to the training partition.

For matched statistical comparisons, differences between each comparator and the full CDR-GNN were calculated within each of the ten common random seed/split pairs. Ninety-five percent confidence intervals were estimated by 10,000 bootstrap resamples of the paired seed-wise metric differences, with the 2.5th and 97.5th percentiles used as the interval bounds. Each difference is reported as comparator minus full CDR-GNN. To assess the potential influence of cohort-derived neuroendocrine prior information, we performed an additional ablation analysis in which all 11 Class N annotations derived from Boullier et al. were removed from the CDR prior. The corresponding metabolites were not removed from the metabolomic data and remained available to the model as measured plasma concentrations. Only their Class N indicators, directional annotations, and CDR weighting were disabled.

## 4. Conclusions

We developed a Cell Danger Response-informed heterogeneous graph framework for three-class Control/NSCLC/NEN discrimination and for the more focused NSCLC-versus-NEN comparison using targeted plasma metabolomics. Comparator and ablation analyses showed that the principal predictive signal was carried by the measured metabolomic features. The CDR prior did not produce a measurable improvement over the otherwise matched concentration-weighted GNN, and its contribution in the present implementation is therefore interpreted as a biologically structured framework for organizing directional metabolite and pathway-level interpretation.

Several limitations should be considered. The primary GNN evaluation was transductive, with validation and test labels excluded from optimization but unlabeled patient features retained in the shared graph. In strict inductive sensitivity analyses, three-class accuracy and macro-F1 were 0.782±0.204 and 0.733±0.207, respectively, while NSCLC-versus-NEN accuracy and F1 were 0.853±0.239 and 0.677±0.370. Although mean AUROC remained high (0.947±0.031 and 0.942±0.128, respectively), threshold-dependent performance was lower and substantially more variable than in the primary transductive analysis.

The cohort is single-center and retrospective, and no independent external validation cohort was available. Substantial between-group differences were also observed in smoking history, pack-year exposure, age, and other clinical characteristics. Although the metabolomics-only models substantially outperformed the clinical-only models, residual clinical confounding cannot be excluded. Accordingly, the present study should be interpreted as a proof-of-concept classification analysis. Future work should include independent multicenter validation, prospectively collected cohorts, larger subtype-specific NEN cohorts, further control of clinical confounding, and assessment of clinically relevant NSCLC grades and other histopathological subgroups.

## Figures and Tables

**Table 1:** Baseline demographic and clinical characteristics of the study cohort. Values are mean ± standard deviation or *n* (%). SMD denotes standardized mean difference.

Variable	Control	NSCLC	NEN	SMD C–NSCLC	SMD C–NEN	SMD NSCLC–NEN
Age, years	60.7±11.0	65.2±8.1	61.9±9.8	0.46	0.11	0.37
BMI	26.6±4.9	27.1±5.2	27.8±6.0	0.10	0.22	0.13
Pack-years	28.4±20.8	43.2±26.3	41.7±26.2	0.62	0.56	0.06
Male	116 (54.2%)	233 (50.0%)	44 (36.7%)	0.08	0.36	0.27
Female	98 (45.8%)	233 (50.0%)	76 (63.3%)	0.08	0.36	0.27
Current smoker	28 (13.1%)	108 (23.2%)	28 (23.3%)	0.26	0.27	0.00
Former smoker	88 (41.1%)	338 (72.5%)	70 (58.3%)	0.67	0.35	0.30
Ever smoker	116 (54.2%)	446 (95.7%)	98 (81.7%)	1.09	0.62	0.45
Race (White)	209 (97.7%)	399 (85.6%)	111 (92.5%)	0.45	0.24	0.22
Other/unknown race codes	5 (2.3%)	67 (14.4%)	9 (7.5%)	0.45	0.24	0.22

NSCLC stage distribution: 1: 200 (42.9%); 2: 75 (16.1%); 3: 98 (21.0%); 4: 43 (9.2%); 5: 50 (10.7%). Comparable stage information was not available for Control or NEN.

**Table 2:** Three-class (Control/NSCLC/NEN) performance of the CDR-informed M-GNN, reported as mean ± standard deviation with 95% confidence interval across ten random seeds on held-out test patients. All metrics are macro-averaged across the three classes.

Metric	Mean ± SD	95% CI
Accuracy	0.904±0.021	0.889–0.920
Balanced accuracy	0.896±0.036	0.870–0.922
Macro-F1	0.898±0.026	0.879–0.916
Precision	0.903±0.021	0.888–0.918
Recall/Sensitivity	0.896±0.036	0.870–0.922
Specificity	0.940±0.016	0.929–0.952
AUROC	0.962±0.016	0.951–0.973
AUPRC	0.940±0.028	0.920–0.960
MCC	0.831±0.039	0.804–0.859

**Table 3:** Per-class three-class performance (mean with 95% confidence interval across ten seeds). The neuroendocrine class is the most cleanly separated and the heterogeneous Control class is the hardest.

Class	Precision	Recall	F1	Specificity	AUROC	Support
Control	0.877	0.835	0.854	0.956	0.951	43
NSCLC	0.917	0.932	0.924	0.881	0.955	93
NEN	0.917	0.921	0.914	0.984	0.980	24

**Table 4:** Collapsed cancer-versus-control classification performance (Stage 1), obtained by summing the NSCLC and NEN posteriors of the three-class model. Mean ± standard deviation with 95% confidence interval across ten seeds.

Metric	Mean ± SD	95% CI
Accuracy	0.925±0.016	0.914–0.936
Balanced accuracy	0.895±0.027	0.876–0.914
F1	0.949±0.010	0.942–0.957
Precision	0.939±0.018	0.926–0.952
Sensitivity	0.960±0.011	0.952–0.967
Specificity	0.830±0.054	0.792–0.869
AUROC	0.951±0.017	0.938–0.963
AUPRC	0.976±0.014	0.966–0.985
MCC	0.806±0.043	0.776–0.837

**Table 5:** NSCLC-versus-NEN subtyping performance (Stage 2), trained and evaluated on the 586 cancer patients (NSCLC n=466, NEN n=120). Mean ± standard deviation with 95% confidence interval across ten seeds.

Metric	Mean ± SD	95% CI
Accuracy	0.960±0.026	0.942–0.978
Balanced accuracy	0.927±0.047	0.893–0.961
F1 (NEN-positive)	0.898±0.065	0.852–0.945
Precision	0.932±0.074	0.880–0.985
Sensitivity	0.871±0.089	0.807–0.934
Specificity	0.983±0.019	0.969–0.996
AUROC	0.985±0.020	0.971–1.000
AUPRC	0.967±0.040	0.939–0.996
MCC	0.876±0.081	0.818–0.934

**Table 6:** Comparator and ablation performance across ten random seeds. Values are reported as mean ± standard deviation.

Model	Accuracy	F1	AUROC	AUPRC
A. Three-class Control/NSCLC/NEN classification				
Clinical only, elastic net	0.535±0.050	0.477±0.040	0.700±0.026	0.550±0.021
Metabolites only, elastic net	0.885±0.027	0.878±0.030	0.958±0.016	0.931±0.026
Clinical + metabolites, elastic net	0.894±0.017	0.889±0.019	0.962±0.018	0.936±0.028
Clinical + metabolites, random forest	0.859±0.025	0.824±0.048	0.962±0.009	0.934±0.015
Clinical + metabolites, MLP	0.892±0.018	0.885±0.021	0.956±0.020	0.932±0.033
Standard unweighted GNN	0.897±0.025	0.891±0.031	0.962±0.015	0.940±0.029
Concentration-weighted GNN, no CDR	0.897±0.019	0.890±0.025	0.961±0.013	0.936±0.024
GNN + uniform CDR boost	0.893±0.022	0.884±0.028	0.961±0.013	0.936±0.024
GNN + directional prior	0.897±0.021	0.889±0.028	0.961±0.013	0.936±0.024
GNN + CDR node prior only	0.894±0.025	0.887±0.029	0.960±0.015	0.936±0.026
Full CDR-GNN	0.895±0.024	0.888±0.028	0.960±0.015	0.936±0.026
Full CDR-GNN, edge-only metabolites	0.613±0.041	0.505±0.043	0.685±0.026	0.531±0.026
B. NSCLC-versus-NEN classification				
Clinical only, elastic net	0.786±0.031	0.254±0.084	0.620±0.028	0.359±0.059
Metabolites only, elastic net	0.968±0.014	0.919±0.037	0.987±0.012	0.949±0.043
Clinical + metabolites, elastic net	0.963±0.015	0.906±0.037	0.984±0.014	0.945±0.042
Clinical + metabolites, random forest	0.875±0.020	0.551±0.109	0.983±0.011	0.954±0.024
Clinical + metabolites, MLP	0.958±0.022	0.889±0.057	0.967±0.031	0.942±0.046
Standard unweighted GNN	0.966±0.020	0.916±0.051	0.975±0.025	0.958±0.038
Concentration-weighted GNN, no CDR	0.966±0.019	0.914±0.050	0.982±0.021	0.966±0.036
GNN + uniform CDR boost	0.966±0.018	0.914±0.047	0.982±0.021	0.966±0.036
GNN + directional prior	0.966±0.019	0.914±0.050	0.980±0.023	0.966±0.036
GNN + CDR node prior only	0.964±0.023	0.907±0.061	0.980±0.023	0.962±0.038
Full CDR-GNN	0.964±0.023	0.907±0.061	0.980±0.023	0.962±0.038
Full CDR-GNN, edge-only metabolites	0.655±0.102	0.318±0.066	0.589±0.059	0.339±0.063

**Table 7:** Paired bootstrap comparison of GNN variants with the full CDR-GNN. Differences are calculated as comparator minus full CDR-GNN across the same ten random seeds. Values are mean paired differences with 95% bootstrap confidence intervals based on 10,000 resamples.

Model	ΔF1 (95% CI)	ΔAUROC (95% CI)	ΔAUPRC (95% CI)
A. Three-class Control/NSCLC/NEN classification			
Standard unweighted GNN	0.0037 (−0.0082, 0.0161)	0.0013 (−0.0026, 0.0058)	0.0039 (−0.0041, 0.0124)
Concentration-weighted GNN, no CDR	0.0023 (−0.0029, 0.0067)	0.0004 (−0.0020, 0.0027)	0.0005 (−0.0039, 0.0044)
GNN + uniform CDR boost	−0.0030 (−0.0118, 0.0042)	0.0004 (−0.0021, 0.0028)	0.0006 (−0.0041, 0.0046)
GNN + directional prior	0.0011 (−0.0038, 0.0058)	0.0005 (−0.0018, 0.0026)	0.0005 (−0.0035, 0.0042)
B. NSCLC-versus-NEN classification			
Standard unweighted GNN	0.0085 (−0.0251, 0.0457)	−0.0052 (−0.0092, −0.0018)	−0.0036 (−0.0159, 0.0075)
Concentration-weighted GNN, no CDR	0.0061 (−0.0061, 0.0234)	0.0016 (−0.0006, 0.0056)	0.0042 (−0.0008, 0.0119)
GNN + uniform CDR boost	0.0064 (−0.0050, 0.0234)	0.0018 (−0.0003, 0.0058)	0.0044 (−0.0006, 0.0122)
GNN + directional prior	0.0061 (−0.0061, 0.0234)	0.0000 (−0.0005, 0.0004)	0.0037 (−0.0009, 0.0111)

Positive differences indicate higher performance of the comparator relative to the full CDR-GNN; negative differences indicate higher performance of the full CDR-GNN. Boldface indicates a 95% bootstrap confidence interval that excludes zero.

**Table 8:** Lung-cancer directional metabolite priors used for CDR-informed patient–metabolite edge weighting. All listed metabolites received the same edge boost of 1.5. Class membership determined whether elevated or decreased standardized deviations were emphasized.

Metabolite	Class	Direction	Edge Boost	Supporting Reference
kynurenine	A	Elevated	1.5	[30]
phenylalanine	A	Elevated	1.5	[31]
glutamic acid	A	Elevated	1.5	[32]
carnitine (C0)	A	Elevated	1.5	[4]
beta-hydroxybutyric acid	A	Elevated	1.5	[4]
SM C24:1	A	Elevated	1.5	[33]
proline	A	Elevated	1.5	[34]
lactic acid	A	Elevated	1.5	[34]
alanine	A	Elevated	1.5	[35]
tryptophan	A	Elevated	1.5	[34]
tyrosine	A	Elevated	1.5	[34]
leucine	A	Elevated	1.5	[36]
isoleucine	A	Elevated	1.5	[36]
serine	A	Elevated	1.5	[37]
pyruvic acid	A	Elevated	1.5	[38]
citric acid	B	Decreased	1.5	[35]
glutamine	B	Decreased	1.5	[34]
taurine	B	Decreased	1.5	[34]
betaine	B	Decreased	1.5	[39]
LysoPC a C20:3	B	Decreased	1.5	[40]
threonine	B	Decreased	1.5	[34]
glycine	B	Decreased	1.5	[34]
choline	B	Decreased	1.5	[41]
valine	B	Decreased	1.5	[42]
histidine	B	Decreased	1.5	[43]
glucose	B	Decreased	1.5	[44]
5-hydroxyindoleacetic acid	N	Elevated	1.5	[6]
C10:1	N	Decreased	1.5	[6]
C10:2	N	Elevated	1.5	[6]
C5DC	N	Elevated	1.5	[6]
fumaric acid	N	Decreased	1.5	[6]
LysoPC a C16:0	N	Decreased	1.5	[6]
LysoPC a C18:0	N	Decreased	1.5	[6]
LysoPC a C18:2	N	Decreased	1.5	[6]
N-acetylputrescine	N	Elevated	1.5	[6]
PC aa C40:2	N	Decreased	1.5	[6]
uric acid	N	Decreased	1.5	[6]

## Data Availability

The primary plasma metabolomics dataset is proprietary and held under ethics restrictions by BioMark Diagnostics Inc. and the IUCPQ-UL biobank; it cannot be publicly deposited. The graph-knowledge-based annotations (metabolite–disease associations and pathway memberships) are derived from the publicly available Human Metabolome Database (HMDB; https://hmdb.ca), and the CDR directional priors are drawn from the published literature as cited in Table 8. The code is available from the corresponding author upon reasonable request and subject to institutional review.

## Abbreviations

The following abbreviations are used in this manuscript:

APUD	amine precursor uptake and decarboxylation
AUPRC	area under the precision–recall curve
AUROC	area under the receiver operating characteristic curve
CDR	Cell Danger Response
CI	confidence interval
COPD	chronic obstructive pulmonary disease
F1	F1 score (harmonic mean of precision and recall)
GNN	graph neural network
GraphSAGE	graph sample and aggregate (inductive graph convolution)
HMDB	Human Metabolome Database
HPLC-MS/MS	high-performance liquid chromatography–tandem mass spectrometry
IUCPQ-UL	Institut universitaire de cardiologie et de pneumologie de Québec–Université Laval
LCNEC	large-cell neuroendocrine carcinoma
LDCT	low-dose computed tomography
LOD	limit of detection
M-GNN	metabolomic heterogeneous graph neural network (framework)
MCC	Matthews correlation coefficient
NEN	pulmonary neuroendocrine neoplasm
NSCLC	non-small-cell lung cancer
PD-L1	programmed death-ligand 1
ROC	receiver operating characteristic
SCLC	small-cell lung carcinoma
SD	standard deviation
TCA	tricarboxylic acid (cycle)
